# Supplementary material for: Antibiotic Resistance and Presence of Persister Cells in the Biofilm-like Environments in Streptococcus agalactiae
Source: Antibiotics (Basel). 2024 Oct 28;13(11):1014. doi: 10.3390/antibiotics13111014 (PMC11590950; doi:10.3390/antibiotics13111014)

Supplementary Materials: *Article*

Antibiotic Resistance and Presence of Persister Cells in the Biofilm-Like Environments in *Streptococcus agalactiae*

Pamella Silva Lannes-Costa [1], Isabelle Rodrigues Fernandes [1], João Matheus Sobral Pena [1], Brunno Renato Farias Verçoza Costa [2], Marcel Menezes Lyra da Cunha [2], Bernadete Teixeira Ferreira-Carvalho [3] and Prescilla Emy Nagao [1,*]

[1] Laboratory of Molecular Biology and Physiology of Streptococci, Institute of Biology Roberto Alcantara Gomes, Rio de Janeiro State University—UERJ, Rio de Janeiro 20550-013, RJ, Brazil;
pamella.lannes@uerj.br (P.S.L.-C.); fernandes.isabelle@graduacao.uerj.br (I.R.F.);
pena.joao@posgraduacao.uerj.br (J.M.S.P.)

[2] Núcleo Multidisciplinar de Pesquisa UFRJ—Xerém em Biologia, Campus UFRJ—Duque de Caxias Professor Geraldo Cidade, Universidade Federal do Rio de Janeiro, Rio de Janeiro 25240-005, RJ, Brazil;
brunno.vercoza@caxias.ufr.br (B.R.F.V.C.); marcel@caxias.ufrj.br (M.M.L.d.C.)

[3] Departamento de Microbiologia Médica, Universidade Federal do Rio de Janeiro, Rio de Janeiro 21941-902, RJ, Brazil; bernadete@micro.ufrj.br (B.T.F.-C.)

* Correspondence: pnagao@uol.com.br or pnagao@uerj.br; Tel.: +55-(021)2334-0541

Table S1. Viability of *Streptococcus agalactiae* strains.

**Cells**	**UNTREATED**			**PEN**			**CLIN**			**ERY**		
	COH1	GBS9036	GBS85147	COH1	GBS9036	GBS85147	COH1	GBS9036	GBS85147	COH1	GBS9036	GBS85147
Live	3900	3784	874	874	754	266	1912	2050	473	420	378	380
Dead	78	76	52	56	131	25	212	225	38	38	37	42
Viability (%)	98	98	94	94	85	91	90	89	92	91	90	89

Table S2. Primers for detection of antimicrobial resistance genes

Gene	Resistance genes<br>Primer Sequence 5’ - 3’		Amplicon
	Foward	*Reverse*	
*tet*M	GAACTCGAACAAGAGGAAAGC	ATGGAAGCCCAGAAAGGAT	740 pb
*tet*O	AACTTAGGCATTCTGGCTCAC	TCCCACTGTTCCATATCGTCA	519 pb
*lin*B	CCTACCTATTGTTTGTGGAA	ATAACGTTACTCTCCTATTC	944 pb
*mef*A/E	AGTATCATTAATCACTAGTGC	TTCTTCTGGTACTAAAAGTGG	348 pb
*erm*A	GCATGACATAAACCTTCA	AGGTTATAATGAAACAGA	206 pb
*erm*B	CGAGTGAAAAAGTACTCAACC	GGCGTGTTTCATTGCTTGATG	616 pb

Figure S1. DNA bands of antimicrobial resistance genes

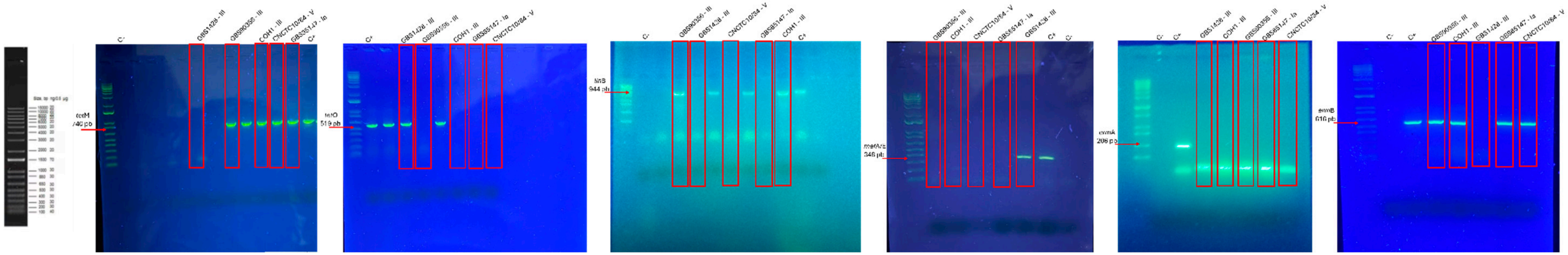

Supplement: Supplementary file 1 [file antibiotics-13-01014-s001.zip › antibiotics-3255331-supplementary.pdf]
